# Intraspecific Functional Trait Variation and Coordination in *Schizachyrium scoparium*

**DOI:** 10.1002/ece3.71635

**Published:** 2025-06-30

**Authors:** Jacob Zeldin, Tessa Dethlefs, Stacy Caeiro, Daniel Puga, Andrea Kramer

**Affiliations:** ^1^ Chicago Botanic Garden, Negaunee Institute for Plant Conservation Science and Action Glencoe Illinois USA; ^2^ Plant Biology and Conservation Northwestern University Evanston Illinois USA

**Keywords:** intraspecific variation, plant functional traits, root traits, Schizachryrium scoparium, trait coordination

## Abstract

Plant functional traits are vital tools in ecological restoration and biodiversity conservation. While functional traits and functional diversity are increasingly being used to inform restoration efforts, challenges remain in the characterization of trait variation in many systems, including within‐species. Likewise, understanding axes of trait variation describing trade‐offs in plant function is important for trait‐based restoration frameworks, yet the degree of coordination between above‐ground functional traits and their below‐ ground counterparts is often unknown. Here, we investigate intraspecific trait variation among five populations of *Schizachyrium scoparium* (little bluestem), a species commonly used for restoration, from different habitat types across a gradient from southern Wisconsin to Northern Illinois. We asked (1) how regional populations of *S. scoparium* differ in their functional traits, (2) how functional trait variation in *S. scoparium* is structured among and within populations, and (3) how above‐ and below‐ground functional traits of *S. scoparium* coordinate and describe axes of functional trade‐offs. We found that populations differed in multivariate trait space, but evidence for differences in individual traits among populations was mixed. Trait relationships with habitat types were idiosyncratic and often misaligned with expectations of plant economic spectra. Variation within populations was as high, or higher, than between populations across traits. We found evidence for weak coordination in several trait pairs, including two above‐ and below‐ground trait combinations, while others appeared to be uncoordinated. Our findings support previous research that trait differentiation can occur at multiple scales, both between and within populations. Extensive within‐population trait variability could be leveraged in trait‐based restoration frameworks targeting intraspecific functional diversity. The lack of strong signals of coordination between above‐ and below‐ground functional traits suggest that sourcing decisions meant to match below‐ground functional traits to recipient restored communities should rely on direct measurement of root traits associated with desired functions rather than above‐ground proxies.

## Graphical abstract

Understanding how traits vary within and between populations of plant species and how axes of trait variation describe trade‐offs in plant function is necessary to fully realize the utility of functional traits in ecological restoration. We investigated intraspecific trait variation among five populations of *Schizachyrium scoparium* (little bluestem), a grass species commonly used for restoration in midwest and prairie regions of the United States. Our findings of extensive within‐population trait variability and signals of coordination in a subset of above‐ and below‐ground functional traits highlight the importance of centering intraspecific trait variation in trait‐based restoration frameworks.

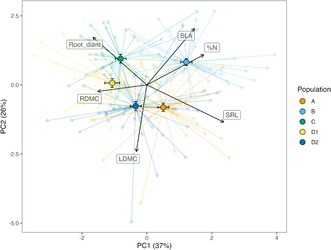

## 1 Introduction

Plant functional traits are crucial factors that drive and are driven by the assembly, stability, and competitive dynamics of plant communities (Májeková et al. 2014; Kunstler et al. 2016; Jones et al. 2019; Luo et al. 2023). Functional traits and their composition in plant communities can directly and indirectly impact ecosystem functions and biodiversity effects (Flynn et al. 2011; Zirbel et al. 2017). As such, the relationships between traits, their composition within plant communities, and the life‐history trade‐offs they represent are increasingly recognized as important tools in the implementation and evaluation of ecological restoration efforts (Funk et al. 2008; Laughlin 2014; Engst et al. 2016; Carlucci et al. 2020). However, challenges remain in building a framework of trait‐based restoration, including gaps in the coverage of studied systems and geographic ranges, questions regarding the proper selection of consequential traits in ecological studies, and the need for more and stronger empirical tests of models linking functional trait composition and ecosystem processes (Funk et al. 2017; Hevia et al. 2017). Further, existing efforts are largely developed using species‐level (interspecific) data on trait variation, despite the important role of intraspecific (within‐species) trait variation on community and ecosystem processes (Bolnick et al. 2011; Violle et al. 2012; Siefert et al. 2015; Funk et al. 2017; Westerband et al. 2021).

Intraspecific trait variation is found both within and between populations (Albert, Thuiller, Yoccoz, Douzet, et al. 2010; Albert, Thuiller, Yoccoz, Soudant, et al. 2010; Mitchell and Bakker 2014; Yoko et al. 2020), and is driven by natural selection, gene flow, and/or random genetic drift (Loveless and Hamrick 1984; Linhart and Grant 1996; Westerband et al. 2021). When populations are distributed across differential biotic or abiotic conditions, natural selection may drive differences in traits: for example, functional traits have been shown to vary between‐ and within‐populations along environmental gradients (Pfennigwerth et al. 2017; Lang et al. 2019; Kühn et al. 2021; Taseski et al. 2021). For species with broad geographic ranges, trait differentiation may correlate with local biotic or abiotic environmental conditions, resulting in increased fitness of populations growing within their home range (i.e., local adaptation). This drives the expectation that trait values will follow geography (Zhou et al. 2013) and geographically proximate populations will have similar traits because they are more likely to be exposed to similar environmental conditions (Joshi et al. 2001).

This expectation is at the heart of restoration seed sourcing policies that favor sourcing germplasm from nearby locations to improve restoration outcomes (Mabry McMullen 2022). However, this expectation is often unsupported: populations may not be adapted to their local conditions (Crespi 2000; McKay et al. 2005), particularly if populations are adapted to historical climate conditions that no longer exist (Anderson and Wadgymar 2020). In situations where populations are not adapted to their site conditions and/or if conditions at the intended restoration site are not similar to any potential source site, different sourcing approaches are increasingly recommended (e.g., regional admixture provenancing; Bucharova et al. 2019). These alternative approaches are further supported by findings of variation in traits within populations on par with variation between populations (Zeldin et al. 2020). Variation within populations can result from localized genetic processes as well as factors like plastic responses to small‐scale environmental variation, stochastic developmental plasticity, or random fluctuations (Scheiner 1993; Baythavong 2011; Herrera 2017). Taken together, the many potential sources of trait variation and uncertainty around the presence and extent of local adaptation highlight the importance of considering trait variation when making germplasm sourcing decisions for restoration.

One key challenge in applying intraspecific functional trait variation to inform restoration germplasm sourcing is identifying how it varies across multiple axes of plant function. Understanding the distribution of functional traits and plant strategies among and within species can be used to inform restoration efforts and target specific ecosystem functioning outcomes (Laughlin 2014). Traits corresponding to different plant organs (i.e., roots and shoots) and aspects of physiology may vary along one or more dimensions, which can in turn influence where plants fall along axes of plant strategies and function (Reich 2014; Díaz et al. 2016). These axes are often described in terms of trade‐offs related to the return on investment in nutrients and carbon (economic spectra; Wright et al. 2004; Roumet et al. 2016). Axes of life‐history trade‐offs are often documented in above‐ground traits and used to place plants along a continuum of acquisitive to conservative life‐history strategies. For example, plants with high specific leaf area (large thin leaves) represent an acquisitive strategy of fast assimilation rates. This is often negatively related to leaf dry matter content, a trait representing a conservative strategy in plants with long‐lived and stress tolerant leaves (e.g., Gorné et al. 2020). Variation in root traits such as specific root length and root diameter may describe similar trade‐offs between resource acquisition and carbon investment or root lifespan (McCormack et al. 2012), leading to efforts to link trait syndromes above‐ and below‐ground. However, variation in below‐ground traits can be multidimensional and driven by different factors than above‐ground traits (Weemstra et al. 2016; Laliberté 2017; Walker et al. 2017). As a result, root traits are not always consistently correlated with leaf traits, especially when above and below‐ground trait coordination is examined within species (Albert, Thuiller, Yoccoz, Douzet, et al. 2010; Umaña and Swenson 2019). This poses challenges for restoration seed sourcing when trait information is lacking below‐ground because root traits can be as important as aboveground traits in predicting performance and survival in certain stressful environments (Garbowski et al. 2020).

In this study, we investigate the structure of, and coordination among, above‐ and below‐ground functional traits in *Schizachyrium scoparium* (little bluestem), an important grass species for restoration efforts in the Great Lakes region of the United States. We use micropropagation to rapidly produce clones of a primarily non‐clonally propagated species, controlling for genetic identity and facilitating a structural analysis of trait variation. We use plant phenotyping to interrogate functional trait values and scales between and within populations sourced from southern Wisconsin and Northern Illinois in order to understand major axes of trait coordination in the species. Specifically, we use this framework to address the following hypotheses:

Hypothesis 1 *Regional populations of S. scoparium differ in their functional trait values above‐ and below‐ground*.

Hypothesis 2 *The structure of trait variation will differ among functional traits and populations of S. scoparium. Trait differences within populations will be comparable to overall trait variation among populations in the region*.

Hypothesis 3 *Above‐ and below‐ground traits will coordinate across populations of S. scoparium*.

The results of this study will yield important insights into trait variability that will inform the selection of plant material for restoration in this species in the Great Lakes region. Results will detail how different sources of variation contribute to the structure of intraspecific trait variation in a dominant grass species and will describe relationships between above‐ and below‐ground functional traits across populations, informing the application of trait‐based frameworks to restoration efforts.

## 2 Methods

### 2.1 Study Species

*Schizachyrium scoparium* (Michx.) Nash (Poaceae) is a widespread, long‐lived, perennial warm‐season C4 grass species native to the United States and southern Canada (Tober and Jensen 2013). *Schizachyrium scoparium* exhibits a caespitose (bunch‐forming) growth form lacking rhizomes, with clonal growth occurring via tiller (ramet) formation proximal to leaf nodes on existing culms (Williams and Briske 1991; Welker and Briske 1992). The species is a common component of various prairie and savanna types in the Great Plains and Midwest regions; however, less common varieties also occur in diverse habitats such as riparian communities in Washington state (Washington Natural Heritage Program 2024) and pine forests of the southeastern United States (Brakie 2016). In southern Wisconsin, *S. scoparium* is a dominant component of dry prairies, dry‐mesic prairies, exposed rock cliffs, and sand barrens (Curtis 1959). In Illinois, *S. scoparium* is among the prominent grass species of dry, sand, gravel, and xeric hill prairies (Corbett and Anderson 2006). Because of its presence in diverse habitat types across a wide geographic range, *S. scoparium* displays coarse‐scale ecotypic variation (Bragg and McMillan 1966; Bruner 1987). In addition to coarse morphological differences across populations and ecotypes, genetic analyses in multiple systems have identified substantial within‐population genetic variation in *S. scoparium* (Huff et al. 1998; Fu et al. 2004). Because of its ubiquity, faunal associations, and affinity for prescribed fire, *S. scoparium* is a commonly used grass species for ecological restoration efforts, especially in the Great Lakes and Central Plains regions (Steinberg 2002; Tober and Jensen 2013).

### 2.2 Seed Collection

We selected five populations of *S. scoparium* across a north–south gradient from southern Wisconsin to northern Illinois. We chose these populations as we were able to obtain permission to collect seed from these locations, and they are characterized by different habitat types and soil properties (e.g., soil type, drainage class: USDA 2024), ranging from dry sand prairies with extremely well‐draining soils to poorly drained, semi‐mesic prairies (Table 1). Each population, apart from the restored Nachusa Grasslands Prairie Potholes population (D2), is a remnant prairie site. Although they were collected along a latitudinal gradient, the populations varied little in their annual precipitation or annual mean temperature (PRISM 2024). Populations were located 50—150 km apart, except the two populations at Nachusa Grasslands that were approximately 1.5 km apart. We collected seeds from 30 maternal plants from each of the populations in September 2021, ensuring that selected maternal lines were > 10 m apart from each other. We dried the collected seed at 15°C and 15% relative humidity for a minimum of 5 days and cleaned the seed, removing all chaff. We randomly selected 10 maternal lines from each population for germination and micropropagation.

**TABLE 1:** Source and habitat information for *Schizachyrium scoparium* seed collections.

Population	Site	State	Habitat type	Soil type	Drainage class	Slope (%)	Annual precip. (in)	Annual mean temp. (*F*)
A	Westport Drumlin	WI	Dry drumlin prairie	Kidder soils	Well‐drained	20–35	37.6	46.6
B	Albany Sand Prairie	WI	Sand prairie	Dickinson sandy loam	Excessively well drained	1–3	37.88	47.4
C	Hosah Park	IL	Dune ecotone	Granby fine sandy loam	Poorly‐drained	0–2	36.26	48.2
D1	Nachusa Grasslands—Isabel's Knob	IL	Dry prairie	Jasper loam	Well‐drained	5–10	38.09	48.4
D2	Nachusa Grasslands—Prairie Potholes	IL	Mesic prairie	Selma loam	Poorly‐drained	0–2	38.15	48.6

*Note:* Populations are ordered and labeled alphabetically north to south, according to latitude. The two sites originating from Nachusa Grasslands were labeled D1 and D2 to indicate their proximity. Soil type, drainage classes, and slopes were obtained from USDA Web Soil Survey (USDA Soil Survey Staff 2024). Annual precipitation and temperatures are 30‐year climate normals (1991–2020) at 800 m resolution obtained from PRISM (PRISM Climate Group 2024).

### 2.3 Germination and Micropropagation

We surface sterilized 20 seeds from each of the selected maternal lines by rinsing the seeds for 60 s in a 70% v/v ethanol solution followed by soaking in a 5% sodium hypochlorite (commercial bleach) solution containing two drops of Polysorbate 20 (Tween) for 15 min and finally rinsing four times with sterile, deionized water. The sterilized seeds were then placed in groups of five on petri dishes containing a germination medium consisting of 8 g/L high gel strength phyto‐agar (A20300, RPI). We then cold stratified the seeds in the dark at 4°C for 30 days to break physiological dormancy and encourage uniform germination (Steinberg 2002), after which the seeds were incubated at 25°C with a 16/8 h day/night photoperiod to initiate germination. Seeds were checked every other day for germination, which was identified as the emergence of both the radicle and cotyledon (scutellum), and visually inspected for contamination.

Overall germination rates were ~70% across populations and contamination was low (< 5%). Seeds with any evidence of microbial contamination were removed from the study. Upon germination, seedlings were transferred to a sterile growing medium composed of 4.3 g/L Murashige and Skoog (MS) basal medium with vitamins (M519, PhytoTech; Murashige & Skoog, 1962), 30 g/L sucrose, 4 g/L phyto‐agar (A20300, RPI), and 1 g/L Gelzan (G3251, PhytoTech). The resulting cultures were incubated at 25°C with a 24‐h photoperiod at 50 μmol s − 1 m − 1 (cool‐white, fluorescent lamps) light intensity. Once we had successfully established one individual seedling from each maternal line, we thinned the cultures to a single seedling per maternal line, resulting in 32 unique genotypes (5–7 genotypes per population, Table 2). As the seedlings grew and produced lateral tillers, we carefully removed the tillers and placed them in new culture vessels on the same growing medium, establishing new plantlets (clones) of each genotype. Resulting cultures were incubated in the same conditions described above, and this process was repeated over the course of 4 months for all cultures until we had obtained sufficient replication (5–9 clones per genotype). No growth regulators or exogenous plant hormones were used during micropropagation.

**TABLE 2:** Sample sizes of functional trait measurements.

Population	Genotypes	Plants	SLA	LDMC	%N	SRL	RDMC	Root diameter
A	7	47	47	46	47	46	47	46
B	7	47	47	47	46	47	47	47
C	5	34	34	34	34	34	34	34
D1	6	41	41	37	41	37	41	37
D2	7	53	48	48	51	52	53	52
Total	32	222	217	212	219	216	222	216

*Note:* Total numbers of unique genotypes and individual plants are detailed for each population along with trait specific sample sizes, accounting for the few instances of missing trait data.

The micropropagated clones (*n* = 222) were de‐flasked in the Spring of 2022 and planted in 128‐cell plug trays containing a well‐draining propagation medium consisting of sphagnum peat moss, perlite, and vermiculite. The clones were acclimated in a fog‐house with elevated humidity and supplemental lighting, located in the Chicago Botanic Garden (Glencoe, IL) production greenhouses, for 4 weeks. Following acclimation, the plants were re‐potted in 11.7 cubic inch (5.5″ depth) conical pots containing a mixture of 40% Turface MVP (calcined, illite clay; Profile Products) and 60% sifted, pulverized topsoil (Menoni & Mocogni, Highland Park, IL). The pots were arranged in trays of 38, and the plants were grown on a bench in a greenhouse in consistent conditions (15°C–19°C daytime, with supplemental lighting, and 14°C–17°C nighttime temperature) at the Chicago Botanic Garden. The placement of the trays on the greenhouse bench was randomized twice a week. The plants were grown for 9 weeks in the greenhouse before being harvested for functional trait measurements.

### 2.4 Functional Trait Measurement

All plants were harvested after growing for 13 weeks *ex vitro*. In July of 2022, plants were removed from their pots and the growing media was gently washed from the root systems. The above‐ground and below‐ground tissues were separated at the crown, and the root systems were temporarily wrapped in damp paper towels, stored in plastic bags, and refrigerated for subsequent root scanning. We measured three leaf functional traits commonly used in plant trait studies that have been shown to represent trade‐offs along an economic spectrum of acquisitive to conservative life‐history strategies and relate to climatic and fertility gradients (Wright et al. 2004, 2005; Reich 2014; Blumenthal et al. 2020); specific leaf area (SLA), leaf dry matter content (LDMC), and percent nitrogen (%N). We also measured three root traits hypothesized to represent an analogous root economic spectrum below‐ground; specific root length (SRL), root dry matter content (RDMC), and root diameter (Reich 2014; Roumet et al. 2016; de la Riva et al. 2021).

Five fully expanded leaves were randomly chosen from each plant for leaf trait measurements and removed at the leaf collar, taking only the lamina and leaving the sheath tissue behind. The selected leaves were scanned at 600 dpi, and the surface area (mm^2^) of each leaf was calculated using ImageJ software (Schneider et al. 2012). The selected leaves were then weighed to retrieve the fresh leaf mass (g), dried at 60°C for 72 h, and re‐weighed to retrieve the dry leaf mass (mg). With these measurements, we calculated specific leaf area (SLA) as the ratio of leaf area to leaf dry mass (mm^2^/mg) and leaf dry matter content (LDMC) as the ratio of leaf dry mass to leaf fresh mass (mg/g). The resulting five leaf trait values for SLA and LDMC were then averaged within each individual. After weighing, the dried leaf samples were pooled per plant and sent to the Danforth Plant Science Center in St. Louis, MO for chemical analysis, where %N was measured via combustion by an elemental analyzer (Elementar vario ISOTOPE cube).

The cleaned root system of each plant was individually scanned at 600 dpi using an Epson Expression 10000XL large‐format flatbed scanner with a transparency attachment, following the protocol from (York 2023). The root systems were floated in a 300 × 420 × 20 mm acrylic box filled with ~400 mL of water for scanning (York 2020). The entire root system of each plant was scanned, though in some cases the root systems needed to be sectioned to fit in the scanning area and ensure that the roots remained submerged. Minor edits were made to the root images to remove the borders of the acrylic box and any shadows from partially submerged roots using the open‐source GIMP software (v. 2.10; (The GIMP Development Team 2019)). Following the scanning procedure, roots were patted dry to remove surface moisture and weighed to retrieve fresh root weight (g). The root samples were then dried at 60°C for 72 h and weighed to obtain dry root mass (mg). These measurements were used to calculate root dry matter content (RDMC) as the ratio of dry root biomass to fresh root biomass (mg/g).

The root scan images were analyzed using the “broken roots” analysis method in RhizoVision explorer (v. 2.0.2; Seethepalli and York 2020). Various settings were tested to analyze root images and segmented images were previewed to assess accuracy. Following testing, all root images were analyzed using the maximum recommended pruning threshold of 20, a non‐root object filter of 1, edge‐smoothing disabled, and an image threshold of 180 to produce the clearest root skeletonization. We extracted total root length (m) and average root diameter (mm) from the RhizoVision analyses and calculated specific root length (SRL, m/g) for each plant by dividing the total root length (m) by the dry root mass (g).

### 2.5 Statistical Analysis

All analyses were carried out in R (v. 4.3.3; R Core Team 2024). To test hypotheses H1 and H2, and to understand how individual functional traits vary between and within populations, we constructed Bayesian hierarchical distributional models using the *brms* package in R (Bürkner 2018). This modeling approach is useful for evaluating variation at multiple sampling levels and permits heterogeneous variance terms, allowing us to test our hypotheses by assessing variation across populations as well as within each population (Mitchell and Bakker 2014). We specified heterogeneous variance components on a per‐population basis and estimated posterior distributions for the population‐level locations (mean trait values) as well as between and within population scales (standard deviations) across hierarchical sampling levels for each functional trait. All traits were scaled to mean 0 and unit variance prior to modeling. Specifically, individual models were built for each functional trait such that:

$$Y_{\mathrm{ijk}}={\text{34𝛽}}_{0}+{\text{34𝜇}}_{\mathrm{ij}}+{\text{34𝑤}}_{\mathrm{ijk}}+{\text{34𝜀}}_{\mathrm{ijk}}$$

where *Y* denotes the trait value for individual *i* of genotype *k* in population *j*. Regional intercepts *𝛽*_0_ were taken to be 0 (as responses were scaled and centered) and randomly varied according to population (*𝜇*_*ij*_) and genotype nested within population (*𝑤*_*ijk*_) with associated standard deviation parameters. Residual errors *𝜀*_*ijk*_ (within‐genotype) were specified with heterogeneous per‐population standard deviations. We specified weakly informative half‐Cauchy priors for the standard deviation parameters centered at 0 with *A* = 3. For each model, we ran Markov Chain Monte Carlo simulations across four chains for 10,000 iterations each with 2500 warm‐up iterations. We validated model convergence through examination of trace‐plots and Rhat values and evaluated model fit with posterior‐predictive checks (comparison of posterior predictions against the empirically observed distributions). We calculated population mean point estimates from the posterior distribution along with 95% and 89% highest density intervals (HDI). We chose to include 95% intervals for reference, however, we rely on the 89% HDI as evaluations of population‐level differences because they are a relatively stable method of describing uncertainty in the posterior distribution (Makowski et al. 2019). We also calculated point‐estimates and 95% and 89% HDI for the posterior between‐population, between‐genotype and residual (within‐genotype) standard deviations for each trait in each population to evaluate the components of the regional trait variation. Trait values from all 222 plants were used to build the models except for cases where trait measurements were missing for some individual plants, leading to marginally smaller sample sizes (*n* = 212–222, Table 2).

In order to test H3 and identify potential axes of functional trait trade‐offs, visualize trait coordination, and assess how populations vary in multivariate trait space, we performed a principal component analysis (PCA) using the *prcomp* function in *R. Prior* to running the PCA, we scaled the six functional traits to mean 0 and unit variance. We evaluated the significance of the PCA, each principal component, and the contribution of each variable to the principal components with permutational and bootstrap tests using the *PCAtest* package (Camargo 2022). We found the PCA and the first two components to be significant (*ψ* = 3.01, *φ* = 0.32, *p* < 0.001), explaining 63% of the variation in the data. We then calculated the population centroids and trait contribution loadings and visualized the results in an ordination. We followed the PCA with an evaluation of coordination between pairs of functional traits using a Bayesian multilevel multivariate correlation model with the *brms* package in R. We specified a global model with a multivariate response including all six functional traits bound using the “mvbind” function in the *brms* package. We specified an intercept only fixed effect structure along with population level random intercept, within‐population correlation, and pairwise residual correlation terms. With partial pooling from the multilevel model, and in the absence of fixed predictor variables, we interpret the residual correlation terms as indicators of trait coordination across the regional pool while considering the relatedness of observations expected within‐populations. We used default, uninformative priors on all model parameters with the addition of an LKJ prior on the residual correlation terms (Lewandowski et al. 2009). We ran Markov Chain Monte Carlo simulations across four chains for 10,000 iterations each with 2500 warm‐up iterations and validated the model with examination of trace‐plots, Rhat values, and posterior‐predictive checks. We extracted point estimates and 95% and 89% HDI of the regional correlation terms for each pairwise trait combination from the posterior distribution.

## 3 Results

### 3.1 Population‐Level Trait Differences

**FIGURE 1:**
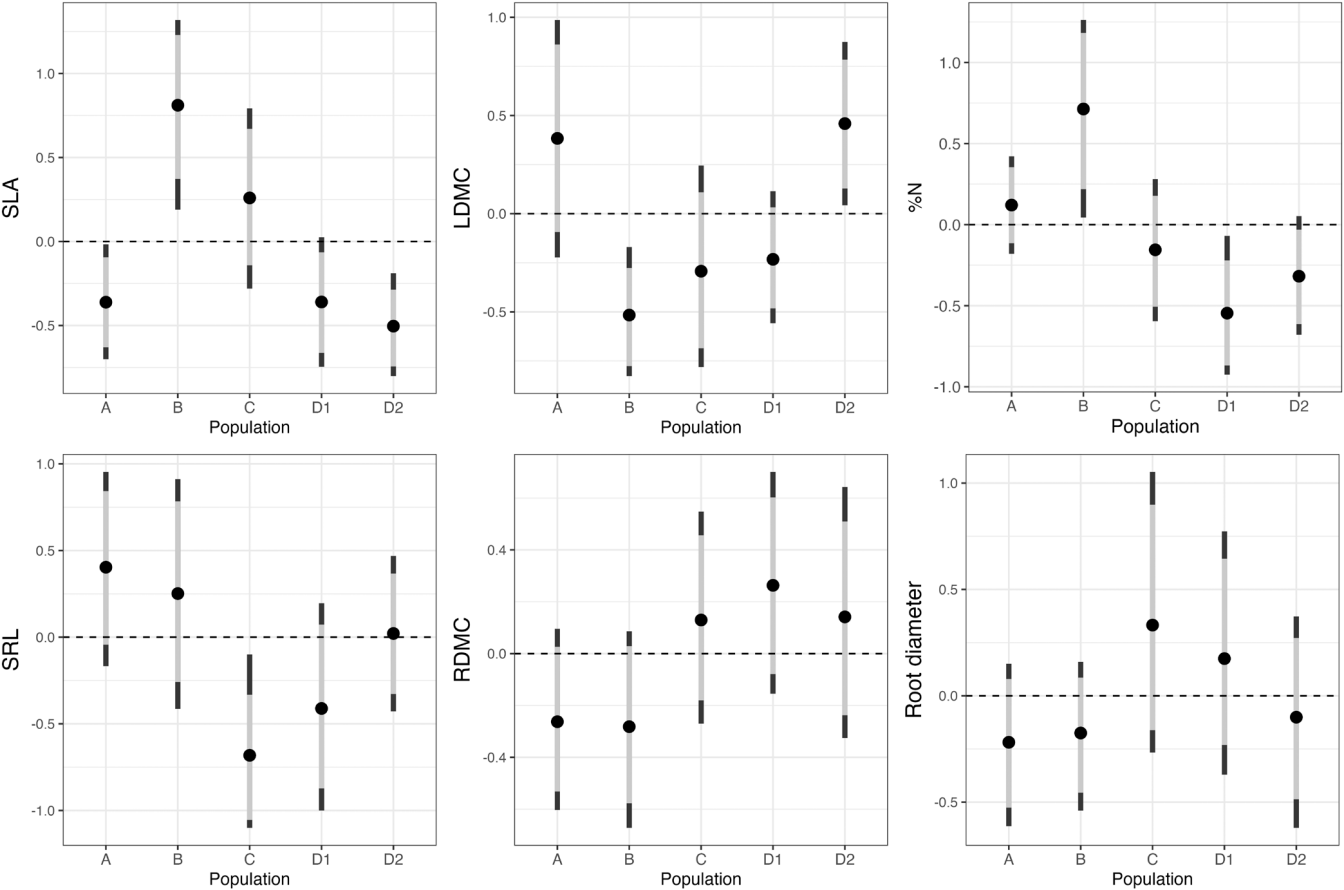
Population trait means across functional traits. Trait means, along with 89% (gray bars) and 95% (black bars) HDIs, were extracted from draws of 10,000 samples of the posterior distribution of the Bayesian hierarchical distributional models for each of the *S. scoparium* populations. Point estimates and HDIs are plotted alongside the overall regional means (regional mean = 0 for scaled responses, dashed lines) as estimates of the population effect sizes.

Trait variation among populations was trait specific, with some traits displaying high differentiation and others very little, providing mixed support for H1 (Figure 1 and Appendix 1). SLA was similar in the northernmost (A) and two southernmost (D1 and D2) populations, all displaying trait values lower than the regional average. SLA in population B was considerably higher than the regional average, and population C overlapped with the regional mean. Variation in LDMC followed a contrasting pattern, where the northernmost (A) and one of the southernmost (D2) populations exhibited high LDMC values, with the 89% HDI for population D2 lying outside of the regional mean. The remaining populations (B, C, and D1) displayed lower LDMC values, though only population B showed strong evidence of deviation, with an HDI fully outside of the regional mean. HDIs of %N overlapped across all five populations; however, %N was higher than the regional average in population B and lower in the two southern populations (D1, D2). SRL values were low in population C relative to the regional mean and all other populations, save D1, where HDIs overlapped. Population A had higher SRL values, only narrowly overlapping the regional mean. We did not find strong differences among population means of RDMC, though the two northern populations (A and B) had low RDMC values, only narrowly overlapping the regional mean. Root diameter was lowest in the two northern populations and population D2; however, HDIs were overlapping among all populations and the regional mean.

### 3.2 Structural Components of Trait Variation

**FIGURE 2:**
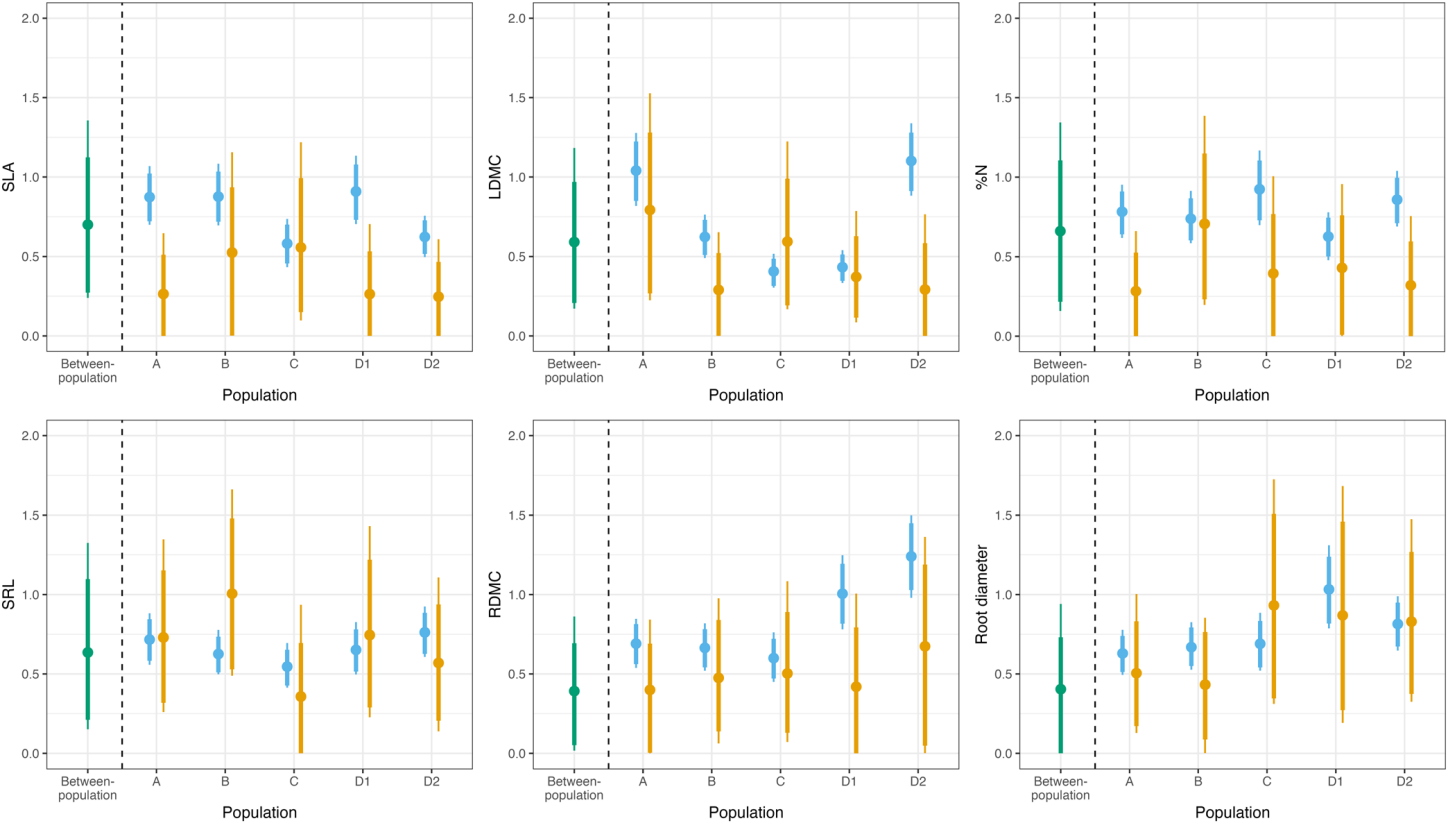
Variation components across populations and traits. Variance components are displayed as between‐population (green), between‐genotype (orange) and within‐genotype (blue) standard deviations (*y*‐axes). Between‐ and within‐genotype standard deviations were estimated individually for each population across the six functional traits. Values displayed as point estimates (means) and HDIs (89% and 95%) drawn from 10,000 samples of the posterior distribution of the Bayesian hierarchical models.

Trait variation within‐populations was generally as high, or higher, than between‐population variation, supporting H2 (Figure 2). Point estimates of the between‐population components of the regional trait variation (*𝜎*) ranged from 0.35–0.72 (Appendix 2), with the highest variation observed in SLA, SRL, and LDMC (*σ* = 0.72, 0.65, and 0.6, respectively). Point estimates of between‐genotype standard deviations were variable across populations in most traits, however, overlapping HDIs indicated a lack of certainty in these differences. Within‐genotype variation, meanwhile, was population dependent and evidence for differences between populations (non‐overlapping HDIs) were observed in within‐genotype standard deviations across all traits except SRL, where the within‐genotype standard deviation was equivalent among populations. Within‐genotype variation was narrowly higher than between‐genotype variation for RDMC across all populations, while the two sources of variation were generally equivalent in root diameter and SRL, exhibiting only minor fluctuations in magnitude according to population. In contrast, among‐ and within‐genotype components of variation in the leaf traits SLA, LDMC, and %N were idiosyncratic and population dependent. Within‐genotype variation was markedly higher than between‐genotype variation for SLA in populations A and D1, for %N in population A, and for all three leaf traits in population D2. The remaining population × trait combinations displayed overlapping HDIs for the between‐genotype and within‐genotype variance components.

### 3.3 Multivariate Responses and Trait Coordination

**FIGURE 3:**
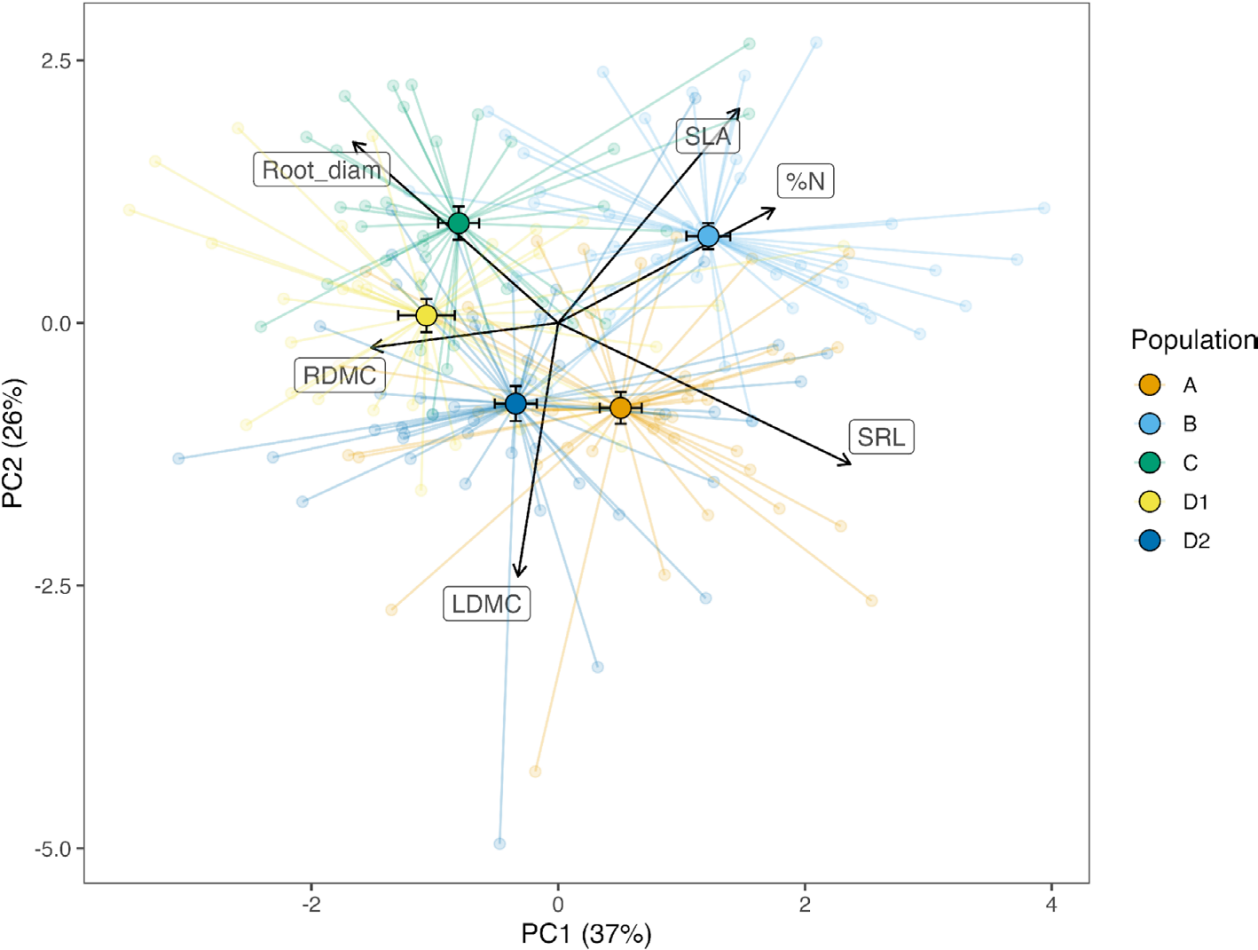
Results of the principal component analysis. Individual scores and population centroids (± std. error) are plotted in multivariate space along the first two principal components, explaining 63% of the variation among six functional traits. Individual plant observations are connected to their respective population centroid to visualize the trait dispersion within populations. Loading vectors are displayed to represent the relative contribution of each trait to the principal components.

The PCA explained 63% of the observed variation across two principal components (PC1 = 37%, PC2 = 26%) with trait loadings indicating possible axes of life history trade‐offs (Figure 3). %N, SRL, SLA, root diameter, and RMDC significantly contributed to the first principal component (permutational tests, loadings = 0.44, 0.59, 0.37, −0.42, and −0.38, respectively). SLA and LDMC significantly contributed to the second principal component (permutational tests, loadings = 0.51 and −0.60, respectively). Population centroids were non‐overlapping overall and visual examination identified three potential groups: populations D1 and C trend toward a conservative root trait syndrome (lower values on PC1), populations D2 and A trend toward a conservative leaf trait syndrome (lower values on PC2), and population B trends toward an acquisitive leaf and root trait syndrome (higher values on PC1 and PC2).

**FIGURE 4:**
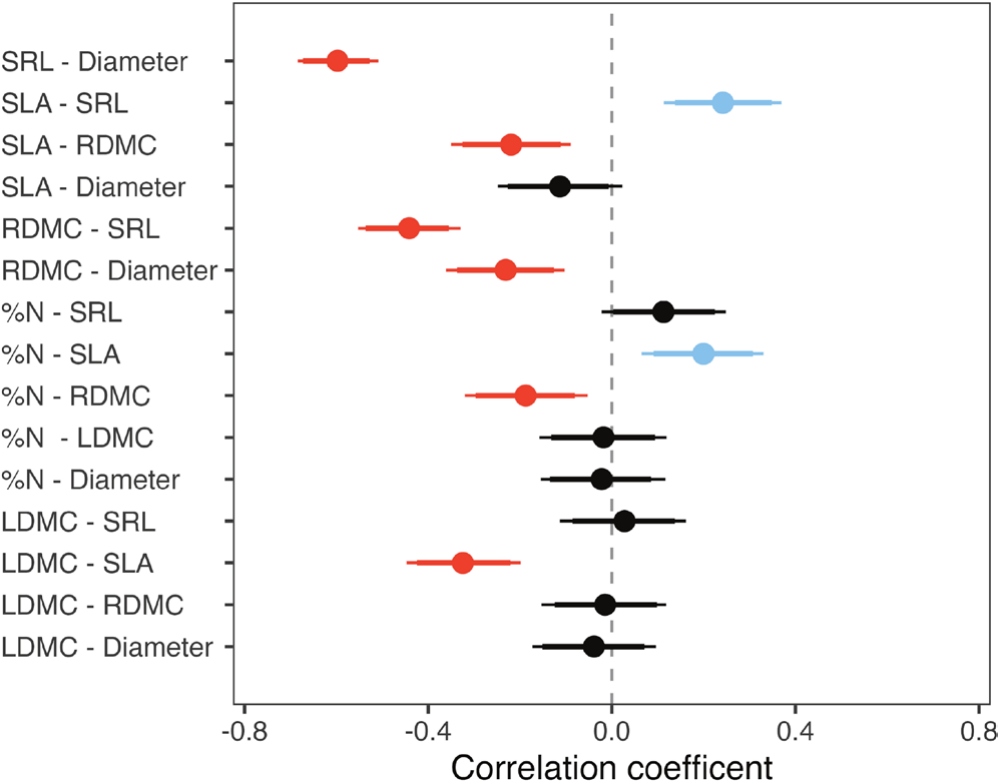
Correlation coefficients from multivariate models of trait coordination. Point estimates and HDIs (89% and 95%) drawn from 10,000 samples of the posterior distribution are displayed for each pairwise trait combination. Red points indicate negatively correlated trait pairs, blue points indicate positively correlated trait pairs, and black points indicate trade pairs that are estimated to be uncorrelated (HDIs cross 0).

Analysis of trait coordination did not fully support H3. There was evidence of weak to moderate trait coordination among 8 of the 15 possible trait combinations (posterior HDI of residual correlation coefficients did not cross 0). Only two of these combinations, SLA‐ SRL (*𝜌* = 0.24, HDI [0.13, 0.35]) and SLA—RDMC (*𝜌 =* −0.22, HDI [−0.32, −0.11]) explained coordination between above‐ and below‐ground traits (Figure 4). We observed a weak positive correlation between %N and SLA (*𝜌* = 0.28, HDI [0.19, 0.38]), and the remaining correlations were negative and within the same strata, in line with expectations of the economic spectrum (Figure 4; Appendix 3) with the exception of RDMC and root diameter, two traits expected to describe conservative life‐history strategies, which were found to be negatively correlated (*𝜌 =* −0.23, HDI [−0.33, −0.13]).

## 4 Discussion

Our results provide mixed evidence for differentiation in functional traits among populations of *Schizachyrium scoparium* in the Southern Wisconsin/Northern Illinois region coupled with considerable and structurally complex within‐population variation. Population centroids appeared segregated in multivariate trait space indicating relatively unique trait syndromes among the sampling locations. These dissimilarities were not clearly explained by geographic distance among populations (our two closest populations clustered relatively far apart while our most distant populations were the most similar in measured trait space) and appear to be driven by multiple axes of trait variation. Within‐population variance components were as high, or higher, than between‐population variation across the majority of traits, with variable and population specific contributions of between‐ and within‐genotype to overall intraspecific trait variation.

### 4.1 Population‐Level Trait Differences

The two southernmost populations, D1 and D2, are located approximately 1.5 km apart in the same managed landscape‐scale restoration (Nachusa Grasslands) and were notably distinct in the location of their central trait syndromes in multivariate space. Though expected to be larger at landscape or regional scales, genetic and phenotypic differentiation can occur at small, local scales (Leimu and Fischer 2008) and considerable trait differentiation has been demonstrated in other native C4 grasses among populations in close proximity (Casler 2005). While we cannot establish local adaptation in the present study, our findings of trait differentiation at local scales indicate the possibility of adaptation to population‐specific environmental conditions. Local adaptation has been shown to occur at fine scales in open, wind‐pollinated grass species (Hamann et al. 2016) and in the presence of high gene flow and phenotypic plasticity (Gonzalo‐Turpin and Hazard 2009). Alternatively, the differences in trait syndromes between the two Nachusa Grasslands populations may be reflective of the more recent restoration of the Prairie Potholes unit (population D2, seed addition 1990–2006, Friends of Nachusa Grasslands 2025). The comparatively lower residence age of this population could have resulted in a mismatch between functional traits and local environmental conditions. Follow‐up population genetic and reciprocal transplant studies could test specific hypotheses of local adaptation in this system.

Interestingly, we found that populations A and D2 shared the most similar trait composition even though they originate from either end of the latitudinal gradient and are found on different soil types with different drainage classes. The causes of convergent trait values in these populations are not apparent, with little discernible similarities in broad soil conditions or habitat type. However, there may be cryptic variables we cannot readily account for that could select for similar trait syndromes. For example, soil microbiomes may play a role in shaping patterns of local adaptation, especially in stressful environments (Petipas et al. 2021; Khasanova et al. 2023; Brady and Farrer 2024).

Among single trait contrasts, population leaf trait values showed the strongest divergence from the regional averages. The most defined differences appeared in populations originating from either end of the soil‐drainage gradient where the direction of leaf trait divergence manifested in a direction opposite to expectations: the dry, most well‐draining population (B) exhibited a traditionally acquisitive leaf trait syndrome of low leaf dry matter content (LDMC) and high specific leaf area (SLA) while the mesic, poorly draining population (D2) exhibited opposite, conservative trends. LDMC has been shown to be closely related to plant drought tolerance and inversely related to soil moisture along hydrological gradients (Blumenthal et al. 2020; Májeková et al. 2021; Taseski et al. 2021). Likewise, SLA is expected to be related to water availability, with studies in other systems demonstrating an increase in mean SLA with growing season precipitation (Wright et al. 2005; Dwyer et al. 2014) and a decrease with diminishing water availability (Milla et al. 2008). SLA and LDMC, however, are also impacted by other environmental factors and land‐use history that could influence their trait distributions in our populations. SLA of perennial grass species can be strongly impacted by nitrogen fertilization (Knops and Reinhart 2000) and both SLA and LDMC may respond to historical disturbances, such as grazing and changes in resource availability across taxa (Quétier et al. 2007; Garnier et al. 2007). We present here only coarse climatic and edaphic characterizations of our study populations; however, more detailed information on land‐use histories, plant community composition, and soil fertility could elucidate alternate drivers of functional trait responses in subsequent studies.

High uncertainty in the models led to a lack of confidence in divergence among most root traits. These findings run counter to strong population‐level differences in root traits that might be expected along a geographic and/or environmental gradient (Dawson et al. 2024). Weaker differentiation among populations and shallower divergence from the regional averages in below‐ground traits relative to leaf traits might suggest that stronger selective pressures are being exerted due to resource limitation above‐ rather than below‐ground. Light availability, for example, could drive trait differentiation among populations where light levels vary in response to differential plant community structure and competitive environments. SLA is related to capacity for light interception and can influence community assembly along gradients of light availability, with high SLA plants performing better under low‐light conditions (Zirbel and Brudvig 2020). As such, it may be that trade‐offs in leaf traits are more strongly governed by photosynthetic capacity or other ecophysiological mechanisms rather than water use efficiency in our system.

As with above‐ground traits, root traits may be expected to follow an economic spectrum in which acquisitive traits associate with productive environments and conservative traits associate with more stressful environments (McCormack et al. 2012; Reich 2014; de la Riva et al. 2021). While overlap in the posterior distributions indicate weaker signals of populations differentiation, all three root traits tended to follow a pattern of more acquisitive strategies in populations with more well‐draining soils, running counter to the conservative trait syndrome expected from a root economic spectrum. Similar results were observed in a study investigating functional traits in tropical tree seedlings subjected to drought treatments which found that variation in below‐ground traits ran counter to the anticipated pattern of the root economic spectrum (Asefa et al. 2022). It is possible that more acquisitive root traits could emerge in areas of low or periodic water availability, resulting in trait responses counter to the hypothesized economic spectrum. Zhou et al. 2019, for example, found high SRL among *Leymus chinensis* at the low end of a precipitation gradient in a semi‐arid temperate grassland. This was hypothesized to be a potential phenotypic adaptation to periodic pulses of precipitation where increased root exploration capacity may be required for rapid water acquisition. We did not, however, observe clear patterns in population means of SRL that would suggest that this trait conforms to either hypothesis, and a more thorough investigation of SRL and other root traits across a steeper hydrological gradient would be required to test these mechanisms.

### 4.2 Within‐Population Trait Variation

Structural assays of functional traits in *S. scoparium* revealed high within‐population variation, with significant variation occurring both between and within genotypes. Our findings of broad trait distributions within populations hold promise for informing source selection in restoration efforts. Intraspecific trait variation and diversity can impact ecosystem dynamics such as competitive relationships and community productivity (Bolnick et al. 2011; Li et al. 2017) and could be leveraged to influence restoration outcomes. Wide trait variability within and among genotypes in restoration seed sources could confer functional redundancy and increase stability in plant communities and/or fill vacant niches to maximize resource use and provide barriers to invasion (Merchant et al. 2022). Indeed, significant trait differences between genotypes are expected to contribute to positive relationships between genotypic richness and ecosystem functions (Crutsinger et al. 2007; Abbott et al. 2017; Raffard et al. 2019).

Intraspecific variation in dominant species may be an especially important source of functional diversity because of their outsized share of abundance in plant communities. Our results demonstrating large within‐population trait variability suggest that sourcing from individual populations with substantial trait breadth could extend trait‐based benefits to restorations in our study system. Still, mixing seed sources following a regional admixture approach may extend additional trait diversity in resulting restorations (Bucharova et al. 2019). Field trials and/or simulation studies would be required to test if mixing source populations provides appreciable gains in the trait diversity of restored populations of *S. scoparium* . Likewise, additional trait studies surveying other species and populations are required to evaluate the generalizability of within‐population variation as described here. Experiments manipulating trait variation within species in a restoration setting would also help to test explicit hypotheses regarding the impact of intraspecific variation on ecosystem functioning and restoration outcomes. We demonstrate here the potential for population‐level trait differentiation in some, but not all traits, which could be leveraged for specific functional goals in a trait‐based restoration framework. However, the substantial trait variability within *S. scoparium* populations could mean that sources with mean trait values outside the desired trait targets may harbor sufficient trait diversity to achieve such targets over time through environmental filtering or selection processes. Overall, our findings support the importance of evaluating and including intraspecific trait variation in trait‐based models for achieving targets in ecological restoration (Laughlin 2014).

We did not find a clear pattern of within‐population trait variation by habitat type, rather, we found similarly high variation across all the study populations. Within‐habitat heterogeneity can have a strong positive influence on intraspecific variation in key functional traits (Karbstein et al. 2020). Micro‐site differences in edaphic conditions, for example, could contribute to high variation in traits related to soil exploration and resource acquisition, such as the within‐population variation we observed in SRL and root diameter. At the same time, within‐population genetic diversity, driven by gene flow in this wind‐pollinated outcrossing species could similarly contribute to intraspecific trait variation. We expect genetic diversity to have a slighter impact on trait variation as previous genetic surveys of *S. scoparium* has highlighted remarkable genetic similarity in accessions from across the United States despite the ubiquitous morphological variation in the species (Harris‐Shultz et al. 2015). Follow‐up population genetic and environmental sampling could help disentangle the relative contributions of heterogeneity of abiotic conditions and genetic distance in explaining trait variation within and across population sources.

Functional traits in this study were measured on plants originating from tissue culture and acclimatized and grown for 13 weeks in greenhouse conditions. Plants propagated in tissue culture conditions can be subject to somaclonal variation, giving rise to differential trait expression within clones which could potentially influence trait variation even after removal from culture and subsequent growth in more standard conditions (Karp 1994). The prevalence of instability and mutations leading to somaclonal variation may be amplified by the use of plant growth regulators and/or propagation methods leveraging undifferentiated tissues (Bairu et al. 2011). While such variation is possible in our study, we are confident that the probability was minimized by propagating using meristematic tissues (tillers) and in the absence of exogenous growth regulators. Still, functional trait measurements from micropropagated plants grown indoors in homogenous conditions may not represent the full breadth of trait variation that could occur in field conditions. Follow‐up common garden studies in a field setting with other species using conventional clonal propagation would provide additional clarity regarding how variation in the present study compares to trait variation in a more realistic setting.

### 4.3 Above‐ and Below‐Ground Trait Coordination

We found that several trait pairs were coordinated across our study populations, however the strength of these relationships were weak to moderate. The majority of correlated trait pairs were reflective of established economic trade‐offs in related plant organs (e.g., LDMC and SLA are inversely correlated and positively and negatively related to leaf lifespan, respectively; Westoby et al. 2002; Kitajima and Poorter 2010), while two relationships, SLA‐SRL and SLA‐RDMC, bridged above‐ and below‐ground plant systems. Previous work linking above‐ and below‐ground functional traits across taxa have found contradictory results depending on the flora, lifeform, and biogeography of the system investigated. For example, a strong positive relationship between SLA and SRL was observed among 11 taxa of temperate tree species, a negative relationship was uncovered among xerophytic woody species in the Tibetan Plateau (Li and Bao 2015), and SLA and SRL were not found to be coordinated among grassland and savanna species across various lifeforms (Tjoelker et al. 2005) or among taxa of Australian wet heathlands (Taseski et al. 2021). Results are similarly mixed among studies of intraspecific trait coordination, with others finding a lack of significant correlation between leaf and root traits (Hajek et al. 2013).

Pairwise relationships between leaf and root traits were weak or uncorrelated and above‐ and below‐ground traits were primarily associated with separate axes of variation in multivariate trait space. Traits reflecting below‐ground tissue density may be expected to coordinate with leaf traits that reflect plant economic strategies above‐ground (such as SLA and LDMC) and we found a weak correlation between SLA and RDMC in our study. However, we found that RDMC was uncorrelated with its parallel leaf trait, LDMC. This result indicates potential multidimensionality in root traits leading to the decoupling of economic spectra above‐ and below‐ground, as has been proposed in other systems (Kramer‐Walter et al. 2016). Further, the discrepancy in the strength and direction of leaf and root trait relationships found here and in other studies at the community and intraspecific scales could potentially be explained by root structural classification and environmental gradients. In a large‐scale transect study of intra‐ and interspecific variation of SLA and SRL in Mongolian grasslands, researchers found functional trait values above‐ and below‐ground to vary along an environmental gradient following a general economic spectrum (low SLA/SRL in resource poor environments and vice versa in richer environments) and that SLA‐SRL relationships changed direction with root branching order (Cheng et al. 2016). Similarly, small‐scale heterogeneity in soil properties explained divergent trajectories of leaf and root traits along an elevation gradient in the French Alps (Weemstra et al. 2022).

The lack of strong coordination among above‐ and below‐ground traits, coupled with extensive within‐population trait variation, suggests that plant functional strategies may not be generalizable across leaves and roots at the intraspecific level. The environmental and plant community contexts experienced by each population are likely to result in limitations among different resource pools and place different selective pressures above‐ and below‐ground, demonstrated in the present study by population differentiation in some trait values. Furthermore, the extensive within‐population variation we observed across traits may indicate the prevalence of trait responses to fine‐scale heterogeneity in the environment, which may impact the detection of trait coordination at the population level. Together, these findings suggest that plant strategies below‐ground may not be inferred from strategies above‐ground at small scales, and leaf traits may serve as unreliable indicators of plant strategies below‐ground. As such, sourcing decisions meant to match functional targets to recipient restored communities should include direct measurement of traits associated with the desired function (e.g., measuring root traits to match edaphic conditions) and/or pair multivariate analysis with investigations of pairwise trait relationships that can provide a more holistic view of trait coordination across multiple strata of plant functions.

## 5 Conclusions

Functional trait ecology is a critical frame of reference through which researchers can gain valuable insight into plant community dynamics, ecosystem functioning, and restoration outcomes. Our research contributes to a growing body of work demonstrating the extent of intraspecific trait variation. Moreover, it outlines the structural composition of above‐ and below‐ground functional traits within‐ and among‐populations of a widespread grass species commonly used in prairie restorations. We highlight traits that display population‐level differentiation and may be expected to exhibit unique trait syndromes relative to the regional average as well as provide context to the selection of restoration germplasm by quantifying the range of trait variation that can be expected when sourcing genotypically diverse seed from populations in the Great Lakes region. Our findings of high within‐population trait variability stress the importance of including intraspecific trait variation in a trait‐based restoration framework and suggest additional research is needed to evaluate how seed sourcing strategies impact intraspecific trait diversity in restorations. Finally, weak patterns of trait coordination above‐ and below‐ground, highlight the importance of evaluating traits across multiple axes of plant function when considering traits in restoration efforts.

## Author Contributions

**Jacob Zeldin:** conceptualization (equal), data curation (lead), formal analysis (lead), investigation (lead), writing – original draft (lead), writing – review and editing (lead). **Tessa Dethlefs:** data curation (supporting), investigation (supporting), writing – review and editing (supporting). **Stacy Caeiro:** investigation (supporting), writing – review and editing (supporting). **Daniel Puga:** investigation (supporting), writing – review and editing (supporting). **Andrea Kramer:** conceptualization (equal), investigation (supporting), resources (equal), writing – review and editing (equal).

## Conflicts of Interest

The authors declare no conflicts of interest.

## Data Availability

The data that support the findings of this study are openly available at Data Dryad—DOI: 10.5061/dryad.3xsj3txrs.

## Appendix 1 Estimated Mean Trait Values by Population

**Table:**

Population	Estimate	89% Lower HDI	89% Upper HDI	95% Lower HDI	95% Upper HDI
SLA					
A	−0.361	−0.629	−0.094	−0.701	−0.017
B	0.811	0.372	1.229	0.190	1.318
C	0.259	−0.141	0.670	−0.280	0.793
D1	−0.360	−0.663	−0.064	−0.746	0.026
D2	−0.504	−0.743	−0.286	−0.801	−0.189
LDMC					
A	0.383	−0.093	0.861	−0.223	0.986
B	−0.516	−0.776	−0.277	−0.827	−0.170
C	−0.293	−0.686	0.109	−0.781	0.245
D1	−0.232	−0.483	0.033	−0.558	0.114
D2	0.459	0.128	0.784	0.043	0.875
%*N*					
A	0.105	−0.145	0.353	−0.218	0.429
B	0.746	0.225	1.260	0.042	1.340
C	−0.189	−0.557	0.175	−0.669	0.291
D1	−0.606	−0.952	−0.239	−1.040	−0.089
D2	−0.281	−0.553	0.004	−0.634	0.077
SRL					
A	0.403	−0.043	0.843	−0.167	0.954
B	0.251	−0.259	0.784	−0.413	0.912
C	−0.682	−1.055	−0.332	−1.100	−0.099
D1	−0.412	−0.873	0.073	−1.000	0.196
D2	0.021	−0.328	0.367	−0.428	0.469
RDMC					
A	−0.263	−0.532	0.026	−0.603	0.095
B	−0.282	−0.577	0.029	−0.672	0.086
C	0.130	−0.180	0.457	−0.270	0.548
D1	0.263	−0.079	0.602	−0.155	0.701
D2	0.142	−0.238	0.509	−0.325	0.643
Root diameter					
A	−0.219	−0.526	0.079	−0.613	0.151
B	−0.175	−0.456	0.085	−0.540	0.159
C	0.332	−0.161	0.898	−0.267	1.053
D1	0.175	−0.231	0.645	−0.371	0.773
D2	−0.101	−0.486	0.272	−0.621	0.373

*Note:* Values expressed as point estimates (means) and HDIs (89% and 95%) of the scaled and centered trait responses drawn from 10,000 samples of the posterior distribution of the Bayesian hierarchical models with lower and upper HDIs (89% and 95%).

## Appendix 2 Estimated Between‐ and Within‐P (Between Genotype, Within‐Genotype) Variation Expressed as Standard Deviations by Population

**Table:**

Variance component	Population	Estimate	89% Lower HDI	89% Upper HDI	95% Lower HDI	95% Upper HDI
SLA						
Between‐population	—	0.719	0.272	1.159	0.219	1.408
Between‐genotype	A	0.533	0.001	0.945	0.001	1.177
	B	0.264	0.000	0.512	0.000	0.646
	C	0.568	0.136	0.995	0.094	1.260
	D1	0.265	0.000	0.533	0.000	0.708
	D2	0.247	0.000	0.466	0.000	0.610
Within‐genotype	A	0.877	0.719	1.034	0.695	1.084
	B	0.874	0.721	1.022	0.699	1.069
	C	0.581	0.456	0.701	0.433	0.736
	D1	0.909	0.729	1.076	0.704	1.134
	D2	0.623	0.517	0.727	0.497	0.756
LDMC						
Between‐population	—	0.601	0.206	0.984	0.174	1.222
Between‐genotype	A	0.290	0.001	0.521	0.000	0.653
	B	0.812	0.269	1.323	0.194	1.589
	C	0.606	0.191	1.008	0.156	1.261
	D1	0.373	0.116	0.629	0.083	0.786
	D2	0.293	0.000	0.585	0.000	0.767
Within‐genotype	A	0.623	0.509	0.73	0.49	0.763
	B	1.04	0.85	1.223	0.818	1.278
	C	0.406	0.315	0.486	0.306	0.52
	D1	0.433	0.347	0.513	0.333	0.539
	D2	1.101	0.912	1.28	0.884	1.338
%*N*						
Between‐population	—	0.600	0.211	1.132	0.152	1.408
Between‐genotype	A	0.283	0.000	0.525	0.000	0.661
	B	0.720	0.236	1.176	0.198	1.440
	C	0.405	0.000	0.780	0.000	1.034
	D1	0.434	0.004	0.761	0.000	0.969
	D2	0.320	0.000	0.596	0.000	0.756
Within‐genotype	A	0.783	0.640	0.911	0.617	0.952
	B	0.739	0.602	0.867	0.584	0.914
	C	0.924	0.727	1.103	0.698	1.168
	D1	0.626	0.502	0.745	0.478	0.779
	D2	0.859	0.710	0.996	0.690	1.039
SRL						
Between‐population	—	0.652	0.193	1.105	0.122	1.354
Between‐genotype	A	1.041	0.513	1.537	0.466	1.793
	B	0.741	0.305	1.155	0.243	1.373
	C	0.366	0.000	0.704	0.000	0.960
	D1	0.762	0.274	1.234	0.232	1.504
	D2	0.573	0.206	0.942	0.138	1.119
Within‐genotype	A	0.626	0.51	0.735	0.498	0.777
	B	0.717	0.583	0.844	0.557	0.881
	C	0.546	0.426	0.652	0.413	0.695
	D1	0.652	0.516	0.781	0.497	0.826
	D2	0.762	0.628	0.886	0.607	0.924
RDMC						
Between‐population	—	0.396	0.046	0.693	0.000	0.842
Between‐genotype	A	0.477	0.137	0.840	0.021	0.937
	B	0.400	0.002	0.690	0.000	0.844
	C	0.510	0.121	0.891	0.061	1.095
	D1	0.425	0.000	0.800	0.000	1.020
	D2	0.689	0.038	1.198	0.003	1.407
Within‐genotype	A	0.665	0.541	0.782	0.521	0.818
	B	0.691	0.562	0.814	0.538	0.846
	C	0.6	0.47	0.721	0.45	0.762
	D1	1.005	0.815	1.192	0.78	1.247
	D2	1.24	1.029	1.449	0.979	1.499
Root diameter						
Between‐population	—	0.408	0.001	0.736	0.000	0.952
Between‐genotype	A	0.434	0.083	0.761	0.001	0.855
	B	0.507	0.170	0.831	0.125	1.004
	C	1.012	0.330	1.671	0.270	2.049
	D1	0.911	0.273	1.557	0.150	1.868
	D2	0.844	0.373	1.294	0.324	1.534
Within‐genotype	A	0.669	0.55	0.793	0.527	0.825
	B	0.63	0.512	0.74	0.494	0.778
	C	0.69	0.542	0.834	0.522	0.886
	D1	1.033	0.817	1.238	0.788	1.312
	D2	0.814	0.673	0.949	0.648	0.989

*Note:* Values expressed as point estimates (means) and HDIs (89% and 95%) drawn from 10,000 samples of the posterior distribution of the Bayesian hierarchical models with lower and upper HDIs (89% and 95%).

## Appendix 3 Correlation Coefficients From Multivariate Models of Trait Coordination

**Table:**

Trait pair	Correlation coefficient	89% Lower HDI	89% Upper HDI	95% Lower HDI	95% Upper HDI
LDMC–Root diameter	−0.04	−0.15	0.07	−0.17	0.10
LDMC–RDMC	−0.01	−0.12	0.10	−0.15	0.12
LDMC–SLA	−0.32	−0.42	−0.22	−0.45	−0.20
LDMC–SRL	0.03	−0.09	0.14	−0.11	0.16
%*N*–Root diameter	−0.02	−0.14	0.09	−0.15	0.12
%*N*–LDMC	−0.02	−0.13	0.09	−0.16	0.12
%*N*–RDMC	−0.19	−0.30	−0.08	−0.32	−0.05
%*N*–SLA	0.20	0.09	0.31	0.06	0.33
%*N*–SRL	0.11	0.00	0.22	−0.02	0.25
RDMC–Root diameter	−0.23	−0.34	−0.13	−0.36	−0.10
RDMC–SRL	−0.44	−0.54	−0.35	−0.55	−0.33
SLA–Root diameter	−0.11	−0.23	−0.01	−0.25	0.02
SLA–RDMC	−0.22	−0.33	−0.11	−0.35	−0.09
SLA–SRL	0.24	0.14	0.35	0.11	0.37
SRL–Root diameter	−0.60	−0.67	−0.53	−0.68	−0.51

*Note:* Point estimates of correlation coefficients and HDIs (89% and 95%) drawn from 10,000 samples of the posterior distribution are provided for each pairwise trait combination.
